# Assessment of genetic risk for improved clinical-neuropathological correlations

**DOI:** 10.1186/s40478-020-01033-1

**Published:** 2020-09-10

**Authors:** Barbara E. Spencer, Robin G. Jennings, Chun C. Fan, James B. Brewer

**Affiliations:** 1 Department of Neurosciences, University of California, San Diego, 9500 Gilman Drive, Mail Code 0949, La Jolla, CA 92093 USA; 2 Center for Human Development, University of California, San Diego, La Jolla, CA USA; 3 Department of Radiology, University of California, San Diego, La Jolla, CA USA

**Keywords:** Alzheimer’s disease, Dementia with Lewy bodies, Parkinson’s disease, Polygenic risk, Diagnosis

## Abstract

In the clinical diagnosis of dementia with Lewy bodies, distinction from Alzheimer’s disease is suboptimal and complicated by shared genetic risk factors and frequent co-pathology. In the present study we tested the ability of polygenic scores for Alzheimer’s disease, dementia with Lewy bodies, and Parkinson’s disease to differentiate individuals in a 2713-participant, pathologically defined sample. A dementia with Lewy bodies polygenic score that excluded apolipoprotein E due to its overlap with Alzheimer’s disease risk was specifically associated with at least limbic (transitional) Lewy-related pathology and a pathological diagnosis of dementia with Lewy bodies. An Alzheimer’s disease polygenic score was associated with neuritic plaques and neurofibrillary tangles but not Lewy-related pathology, and was most strongly associated with an Alzheimer’s pathological diagnosis. Our results indicate that an assessment of genetic risk may be useful to clinically distinguish between Alzheimer’s disease and dementia with Lewy bodies. Notably, we found no association with a Parkinson’s disease polygenic score, which aligns with evidence that dementia with Lewy bodies has a distinct genetic signature that can be exploited to improve clinical diagnoses.

## Introduction

In heterogeneous disease cohorts, accurate distinctions between Alzheimer’s disease (AD) and related dementias may improve precision in care delivery and thus lead to better outcomes. In the diagnosis of dementia with Lewy bodies (DLB), distinction from AD is suboptimal and complicated by the frequent co-occurrence of AD neuropathologic changes (NC) with Lewy body (LB) pathology. Patients clinically diagnosed with AD often present with concurrent LB pathology at autopsy, though many studies have attempted to tease apart the differences in clinical presentations to better reflect underlying pathology [2, 6, 9, 14, 21, 25].

Incorporating information about genetic risk into a difficult differential diagnosis may improve clinical-neuropathological correlations. A recently developed AD polygenic hazard score (PHS) is associated with the hallmark Alzheimer’s disease neuropathologic changes (ADNC), neuritic plaques and neurofibrillary tangles. However, the large-scale genetic studies that identify such risk typically rely on clinical diagnoses, which are imperfect proxies for the often mixed underlying pathologies [5, 20]. While the AD PHS has reported associations with LBs [22], it is unclear whether this reflects a shared genetic risk between pathologies, a byproduct of the common presence of LB co-pathology with AD, since the level of ADNC was not controlled for in the analysis, or a lack of specificity in the AD PHS due to the presence of LBs or other mixed pathologies in those clinically diagnosed with AD.

DLB has both clinical features and genetic risk factors that overlap with both AD and Parkinson’s disease (PD). The apolipoprotein E (*APOE*) ε4 allele is the strongest genetic risk factor for late onset AD and is also overrepresented in pure DLB and PD with dementia [24]. The *SNCA*, *GBA*, and *BCL7C/STX1B* genes are implicated in risk for both DLB and PD [8, 16], though the associations at the *SNCA* locus differ between the two [1, 8]. Given the relatively low accuracy of a DLB diagnosis, genetic studies typically examine relatively small, neuropathologically confirmed DLB cohorts [8, 19], with few genome-wide significant variants identified. Then, genetic risk for PD, discovered in well-powered studies, may be better suited to predict the underlying LB pathology in DLB cases. In a pathologically defined cohort we tested the hypothesis that the AD PHS, a DLB polygenic risk score (PRS), and a PD PRS can differentiate individuals with DLB from those who have AD.

## Methods

### Participants

A sample of 437 participants was selected from the Shiley-Marcos Alzheimer’s Disease Research Center (ADRC) of the University of California, San Diego (UCSD). An independent sample of 3982 participants evaluated at other ADRCs was selected from the National Alzheimer’s Coordinating Center (NACC). Inclusion was limited to participants who had undergone genotyping and a neuropathological assessment at autopsy. All data were collected through the NACC uniform data set, minimum data set, or neuropathology data set, except where otherwise specified.

### Pathological diagnosis

The 4419 participants were categorized based on diagnostic criteria for AD, DLB, frontotemporal lobar degeneration (FTD), medial temporal lobe sclerosis (MTLS), and other major pathological diagnoses as follow.

AD: If Thal phase was assessed, an “ABC” score indicating intermediate or high ADNC [11] constituted an AD diagnosis. Otherwise, pathological diagnosis of AD followed NIA-Reagan criteria (i.e., at least Consortium to Establish a Registry for Alzheimer’s Disease (CERAD) moderate and Braak stage III/IV) [12].

DLB: Pathological diagnosis of DLB followed criteria outlined in the fourth consensus report of the DLB Consortium (i.e., requires limbic (transitional) or diffuse neocortical Lewy-related pathology) [14].

MTLS: MTLS (including hippocampal sclerosis) was determined based on pathologist report to the NACC as present or absent.

FTD and other tauopathies: Evidence of FTD with tau pathology (including Pick’s disease, corticobasal degeneration, and progressive supranuclear palsy), FTD and parkinsonism with tau-positive or argyrophilic inclusions, other tauopathies (including tangle-only dementia and argyrophilic grain dementia), FTD with ubiquitin-positive (tau-negative) inclusions, FTD with TDP-43 pathology, and FTD with no distinctive histopathology present or not otherwise specified constituted an FTD pathological diagnosis.

Other Pathological Diagnoses: Cases with “other pathological diagnoses” were excluded. Specifically, in versions 1–9 of the NACC neuropathology data, this constituted evidence of prion-related disorders and other major pathologic disorders (e.g. infectious, immunologic, metabolic, neosplastic, toxic, or degenerative). In version 10, this constituted ALS/motor neuron disease, Pigment-spheroid degeneration/NBIA, multiple system atrophy, prion disease, trinucleotide disease (Huntington disease, SCA, or other), malformation of cortical development, metabolic/storage disorder, leukodystrophy, multiple sclerosis or other demyelinating disease, contusion/traumatic brain injury of any type (acute or chronic), neoplasm (primary or metastatic), infectious process of any type (encephalitis, abscess, etc.), herniation (any site), or other pathologic diagnosis, Down syndrome, AD-related genes (dominantly inherited), FTD related genes (dominantly inherited), or other known genetic mutation. Neuron loss in the substantia nigra was additionally considered except in the case of a DLB pathological diagnosis.

To disentangle the effects of genetic risk on pathology in light of frequently occurring co-pathology, we restricted analysis to individuals who met the above criteria for only AD (n = 1854), DLB (n = 57), or FTD (n = 65) without meeting criteria for any other pathological diagnosis, those who met the criteria for both AD and DLB but no other pathological diagnosis (AD + DLB, n = 455), and those who met the criteria for both AD and MTLS but no other pathological diagnosis (AD + MTLS, n = 182). Also, individuals who did not meet criteria for any of the above pathological diagnoses were included (control, n = 245). This lead to the exclusion of 1561 participants based on pathological criteria, either with mixed pathology inconsistent with the above groups, or with pathology documented in a way that precluded categorization (e.g. LBs present in an unspecified region).

### Clinical diagnoses

Clinical diagnosis was assessed at the final visit before death. Individuals in the control group (who did not meet criteria for any of the pathological diagnoses) were further limited to clinically normal participants without PD, resulting in the exclusion of 68 additional participants. For all other pathologically defined groups, no clinical criteria were imposed except to exclude individuals with PD dementia, following the one-year rule, excluding an additional 14 participants (1 DLB and 13 AD + DLB), and those with a prior clinical PD diagnosis who did not meet pathological criteria for DLB, excluding an additional 63 participants (55 AD, 7 AD + MTLS, and 1 FTD).

### Genetic data

Genetic data for UCSD ADRC participants was accessed through the NACC database or obtained locally. Alzheimer’s disease Genetics Consortium (ADGC) data for participants evaluated at other ADRCs was accessed through the National Institute on Aging Genetics of Alzheimer’s disease Data Storage Site (NIAGADS). For the ADRC, genetic data was preprocessed with PLINK to exclude samples with a missingness rate greater than 10% and to perform strand flips as necessary. Pre-imputation quality controls removed duplicate sites, non-single-nucleotide polymorphism (SNP) sites, monomorphic sites, and SNPs with a call rate < 90%. The imputation was performed using the Michigan Imputation Server [3] with the HRC reference panel [13] (hg19). Post-imputation the data was filtered to exclude genotype calls with an estimated posterior genotype probability < 0.9. For the NACC, imputed genetic data was downloaded directly from NIAGADS.

### AD polygenic hazard score calculation

The PHS was calculated as described for all participants [4]. Briefly, potentially AD-associated SNPs were selected in the International Genomics of Alzheimer’s Project (IGAP) cohort at *p* < 10^−5^. These SNPs were then integrated into a stepwise Cox proportional hazards model using a subset of the ADGC phase 1 genetic data, excluding individuals from the NACC, Alzheimer’s Disease Neuroimaging Initiative (ADNI), and the Religious Orders Study and Rush Memory and Aging Project (ROSMAP). This stepwise procedure identified 31 SNPs that most improved the model prediction. The derived PHS was then validated in independent samples, including ADGC phase 2, NACC, ADNI, and ROSMAP. The PHS used in the current study was calculated for each participant as the vector product of that individual’s genotype for the 31 SNPs and the corresponding parameter estimates from the ADGC phase 1 Cox proportional hazard model, in addition to the *APOE* effects.

### PD polygenic risk score calculation

The PD PRS was calculated for each participant as the vector product of that individual’s genotype for the 90 independent genome-wide significant variants identified by the most recent meta-analysis of PD genome-wide association study (GWAS) data and the corresponding parameter estimates using data from all available studies [15].

### DLB polygenic risk score calculation

The DLB PRS was calculated as the vector product of that individual’s genotype for the 5 independent genome-wide significant variants identified by the first DLB GWAS and the corresponding parameter estimates from the discovery stage [8]. To ensure there was no participant overlap between samples, the DLB PRS was only analyzed in the subset of 2282 participants in the present study who were assuredly not included in the DLB GWAS.

### Statistical analysis

Binary logistic regression was used to examine the relationship between the PD PRS and a clinical diagnosis of PD, controlling for age at death and sex. Clinical and demographic differences between pathologically defined groups were examined using either Welch’s two sample *t* test or Pearson’s Chi squared test as appropriate, corrected for multiple comparisons using the Benjamini–Hochberg procedure. *APOE* ε4 allele frequency was examined across pathologically defined groups using Pearson’s Chi squared test. Pairwise comparisons between groups were corrected for multiple comparisons using the Benjamini–Hochberg procedure.

Multinomial logistic regression models were used to examine the relationship between the AD PHS, PD PRS, or DLB PRS and the pathological diagnosis group, controlling for age at death and sex, and Bonferroni corrected for three comparisons. Given that the *APOE* ε4 allele is a known risk factor for both AD and DLB, multinomial logistic regression models were used to examine the relationship between either the AD PHS or DLB PRS without its *APOE* component weights and the pathological diagnosis group, controlling for age at death and sex.

Ordinal logistic regression models were used to examine the relationship between either the AD PHS, PD PRS, or DLB PRS and AD pathological outcome variables (i.e., Braak stage for neurofibrillary tangles or CERAD score for neuritic plaques), controlling for age at death and sex. Multicollinearity was evaluated. Brant’s test was used to test the proportional odds assumption. Proportional odds models were used except in the case where the proportional odds assumption was violated, in which cases partial proportional odds models were used. Binary logistic regression models were used to examine the relationship between the AD PHS, PD PRS, or DLB PRS and the presence of at least limbic (transitional) Lewy-related pathology, controlling for age at death and sex. For each pathological outcome variable, results were Bonferroni corrected for three comparisons. To examine the effect of the AD PHS and DLB PRS on pathology beyond *APOE*, analyses were repeated using versions of the AD PHS or DLB PRS without its *APOE* component weights.

## Results

In all 4419 participants, regardless of pathological diagnosis, the PD PRS predicted a clinical diagnosis of PD (odds ratio (OR) = 1.52, 95% confidence interval (CI) = 1.16–1.98, *p* = 0.002). 2713 participants were categorized into one of six pathologically defined groups: FTD (n = 64), DLB (n = 56), AD + DLB (n = 442), AD (n = 1799), AD + MTLS (n = 175) or control (n = 177). Clinical and demographic characteristics of these pathologically defined groups are reported in Table 1. Notable in Table 1 is the following: the age at death in each pathologically defined group, with the exception of AD + MTLS, was younger than in the control group, more men were in the DLB and AD + DLB groups than in the other groups, all AD groups (AD, AD + DLB, and AD + MTLS) had worse cognitive impairment than the FTD or DLB groups, and, within AD groups, those with mixed pathology (AD + DLB or AD + MTLS) had worse cognitive impairment than those with only AD.

**Table 1:** Demographics and clinical characteristics split by pathological diagnosis group

	Pathological diagnosis group					
	Control	FTD	DLB	AD + DLB	AD	AD + MTLS
Participants, N	177	64	56	442	1799	175
Women, N (%)	94 (53)	25 (39)	12 (21)^*§¶#^	181 (41)^*¶#^	959 (53)	94 (54)
Age at death, y	83.7 (8.7)	78.5 (11.5)^*#^	80.1 (8.6)^*#^	79.0 (8.5)^*#¶^	80.2 (9.0)^*#^	84.6 (7.8)
Caucasian, N (%)	173 (98)	63 (98)	56 (100)	427 (97)	1736 (96)	171 (98)
Hispanic, N (%)	4 (2)	0 (0)	1 (2)	7 (2)	30 (2)	1 (1)
Education, y	15.4 (2.8)	15.1 (3.0)	15.6 (3.3)	15.0 (3.2)	14.3 (3.3)^*‡§^	14.8 (3.6)
*Final clinical evaluation*						
Time before death, y	2.6 (3.0)	3.0 (3.0)	2.9 (3.5)	3.0 (3.3)	2.5 (2.8)^§#^	3.2 (3.1)
*Primary clinical diagnosis*						
AD, N (%)	0 (0)	22 (34)	19 (34)	340 (77)	1574 (87)	159 (91)
LBD, N (%)	0 (0)	2 (3)	24 (43)	79 (18)	41 (2)	4 (2)
FTD, N (%)	0 (0)	23 (36)	1 (2)	9 (2)	64 (4)	5 (3)
Global CDR	0.1 (0.2)	1.6 (1.2)^*^	1.4 (1.0)^*^	2.1 (0.9)^*†‡^	1.9 (1.0)^*†‡§#^	2.2 (0.8)^*†‡^
CDR-SB	0.1 (0.4)	9.0 (6.8)^*^	7.6 (5.8)^*^	12.5 (5.5)^*†‡^	10.8 (6.1)^*†‡§#^	12.9 (4.9)^*†‡^

Reported as mean (SD) unless otherwise noted. Primary clinical diagnosis of AD included probable or possible AD, LBD included DLB, Lewy body variant of AD, and LBD, and FTD included FTD, Pick’s disease, CBD, PSP, and PPA. FDR adjusted *p* < .05 for differences from *Control, †FTD, ‡DLB, §AD + DLB, ¶AD, or #AD + MTLS based on pairwise Pearson’s Chi squared tests or Welch’s t-tests. Small numbers in certain subgroups prevented the pairwise comparison of primary clinical diagnoses across pathologically defined groups

Abbreviations: DLB, dementia with Lewy bodies; AD, Alzheimer’s disease; FTD, frontotemporal lobar degeneration; MTLS, medial temporal lobe sclerosis; LBD, Lewy body dementia; CDR, Clinical Dementia Rating Scale; CDR-SB, Clinical Dementia Rating Scale Sum of Boxes

*APOE* ε4 allele frequency was different between pathologically defined groups (overall χ^2^ = 174.5, *p* < .001, Table 2). Pairwise Chi squared tests revealed that *APOE* ε4 allele frequency was greater in all AD groups (AD, AD + DLB, and AD + MTLS) and the DLB group than in the control group (FDR adjusted *p* < .05). Additionally, *APOE* ε4 allele frequency was greater in all AD groups than in either the FTD or DLB groups (FDR adjusted *p* < .001).

**Table 2:** *APOE* genotypes and allele frequencies by pathologically defined groups

	N (%)						
	*APOE* genotype frequency						ε4 allele frequency
	2/2	2/3	2/4	3/3	3/4	4/4	
Control	1 (1)	25 (14)	7 (4)	119 (67)	24 (14)	1 (1)	33 (9)
FTD	0 (0)	7 (11)	1 (2)	37 (58)	19 (30)	0 (0)	20 (16)†
DLB	0 (0)	8 (14)	4 (7)	28 (50)	16 (29)	0 (0)	20 (18)*†
AD + DLB	2 (0)	11 (2)	5 (1)	136 (31)	224 (51)	64 (14)	357 (40)*
AD	0 (0)	52 (3)	51 (3)	594 (33)	829 (46)	273 (15)	1426 (40)*
AD + MTLS	0 (0)	8 (5)	5 (3)	58 (33)	83 (47)	21 (12)	130 (37)*

*APOE* ε4 allele frequency was different between pathologically defined groups (overall χ^2^ = 174.5, *p* < .001). FDR adjusted *p* < .05 for differences from *Control, †All AD groups (AD, AD + DLB, and AD + MTLS) based on pairwise Chi squared tests

Abbreviations: DLB, dementia with Lewy bodies; AD, Alzheimer’s disease; FTD, frontotemporal lobar degeneration; MTLS, medial temporal lobe sclerosis; *APOE*, apolipoprotein E

**Fig. 1:**
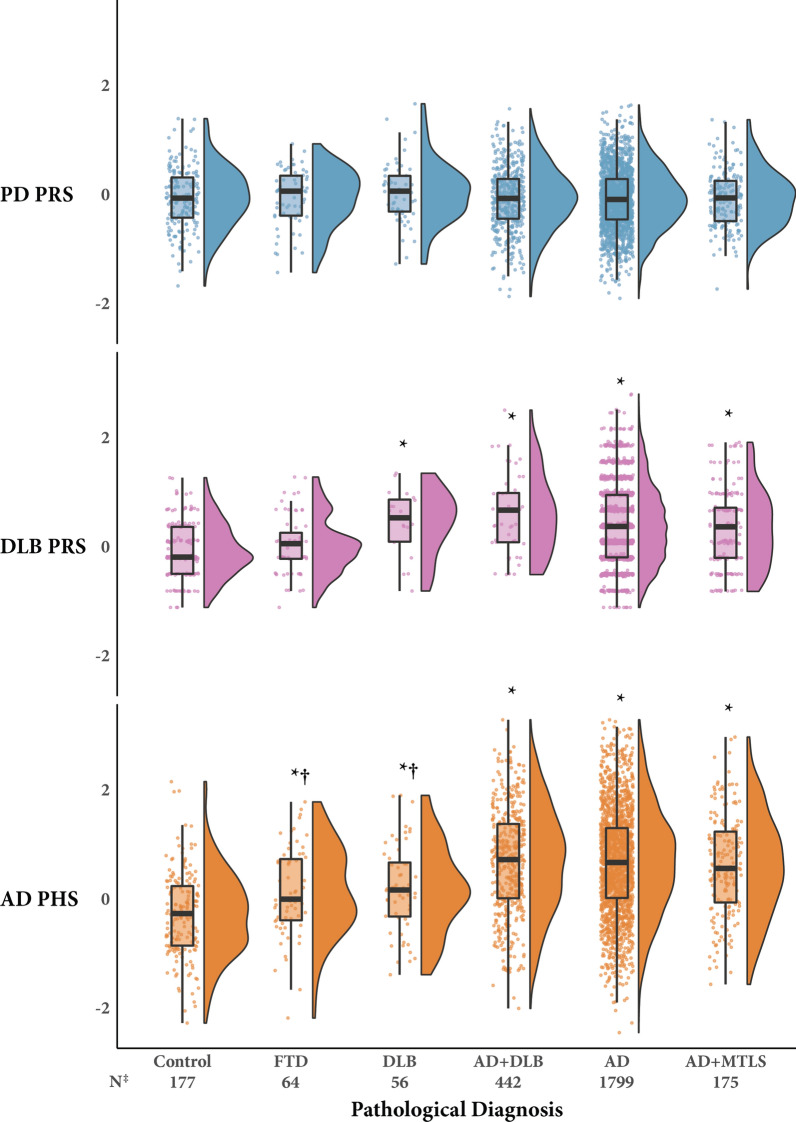
Relationship between polygenic scores and pathological diagnostic categories. Significant difference from: *Control, †All AD groups (AD, AD + DLB, and AD + MTLS) based on Bonferroni corrected multinomial regression models. ‡The number of participants is reported for the AD PHS and PD PRS analyses. The DLB PRS was analyzed in a subset of 2282 total participants. Abbreviations: PD, Parkinson’s disease, PRS, polygenic risk score; DLB, dementia with Lewy bodies; AD, Alzheimer’s disease; PHS, polygenic hazard score; FTD, frontotemporal lobar degeneration; MTLS, medial temporal lobe sclerosis

Higher AD PHS was associated with increased relative risk ratios for the AD, AD + DLB, and AD + MTLS pathological diagnosis groups, compared to the control group (*p* < .001, Table 3; Fig. 1). Higher AD PHS was also associated with increased relative risk ratios for the DLB and FTD groups compared to the control group (*p* < .01), though with lower relative risk ratios than any of the AD groups, as confirmed by non-overlapping confidence intervals. Higher PD PRS was not associated with significant increased relative risk ratios for any pathological group compared to the control group. Higher DLB PRS was associated with increased relative risk ratios for the AD, AD + DLB, AD + MTLS, and DLB groups compared to the control group (*p* < .001).

**Table 3:** Associations between the polygenic scores and pathological diagnostic categories and variables

Pathological diagnosis	AD PHS		PD PRS		DLB PRS	
	*RRR (95% CI)*	*p value*	*RRR (95% CI)*	*p value*	*RRR (95% CI)*	*p value*
*MLR, control as reference*						
FTD	1.60 (1.16–2.21)	.004	1.01 (.60–1.70)	.97	1.24 (.76–2.02)	.40
DLB	1.73 (1.23–2.42)	.002	1.39 (.80–2.41)	.24	3.22 (1.62–6.40)	< .001
AD + DLB	3.05 (2.47–3.77)	< .001	.93 (.67–1.28)	.65	4.47 (2.76–7.22)	< .001
AD	3.06 (2.52–3.71)	< .001	.92 (.69–1.22)	.57	3.08 (2.33–4.09)	< .001
AD + MTLS	3.14 (2.45–4.02)	< .001	.95 (.65–1.39)	.78	2.94 (2.07–4.18)	< .001

	*OR (95% CI)*	*p value*	*OR (95% CI)*	*p value*	*OR (95% CI)*	*p value*
*Braak stage*						
OLR, POM	–	–	0.93 (0.82–1.05)	.24	–	–
OLR, PPOM						
I	2.34 (1.60–3.42)	< .001	–	–	1.89 (1.06–3.39)	.03
II	2.10 (1.76–2.50)	< .001	–	–	2.05(1.52–2.76)	< .001
III	2.28 (1.99–2.62)	< .001	–	–	2.33 (1.88–2.89)	< .001
IV	1.88 (1.68–2.10)	< .001	–	–	1.95 (1.63–2.33)	< .001
V	1.53 (1.40–1.68)	< .001	–	–	1.50 (1.30–1.72)	< .001
VI	1.33 (1.23–1.44)	< .001	–	–	1.34 (1.18–1.51)	< .001
*Neuritic plaque density*						
OLR, POM	–	–	0.85 (0.73–0.99)	.04	–	–
OLR, PPOM						
Sparse	2.69 (2.21–3.26)	< .001	–	–	2.79 (1.95–3.98)	< .001
Moderate	2.42 (2.07–2.84)	< .001	–	–	2.42 (1.88–3.12)	< .001
Frequent	1.71 (1.55–1.88)	< .001	–	–	1.63 (1.41–1.88)	< .001
*Lewy pathology stage*						
BLR	1.03 (0.93–1.14)	.52	1.04 (0.87–1.24)	.66	1.45 (1.04–2.03)	.03

Results of multinomial, ordinal, and binary logistic regression models for each polygenic score and pathological diagnosis group and outcome variable are displayed. For ordinal logistic regressions, proportional odds models were used except in the case where the proportional odds assumption was violated, in which cases partial proportional odds models were used. Effects are reported per unit increase in the polygenic score. Significance was set to *p* < .017, Bonferroni corrected for comparisons across the three polygenic scores

Abbreviations: RRR, relative risk ratio; OR, odds ratio; MLR, multinomial logistic regression; OLR, ordinal logistic regression; POM, proportional odds model; PPOM, partial proportional odds model; BLR, binary logistic regression PD, Parkinson’s disease, PRS, polygenic risk score; DLB, dementia with Lewy bodies; AD, Alzheimer’s disease; PHS, polygenic hazard score; FTD, frontotemporal lobar degeneration; MTLS, medial temporal lobe sclerosis

Without the *APOE* ε4 or ε2 dosage weights, we observed similar results for the AD PHS, which was associated with increased relative risk ratios for the AD (1.86 95% CI [1.47–2.37]), AD + DLB (1.83 [1.39–2.40]), AD + MTLS (1.91 [1.37–2.65]), DLB (2.03 [1.26–3.26]), and FTD (1.78 [1.14–2.78]) groups compared to the control group. Without the *APOE* component weight, the specificity of the DLB PRS emerged, as it was only associated with increased relative risk ratios for the DLB (3.58 [1.20–10.66]) and AD + DLB (3.15 [1.47–6.77]) groups compared to the control group.

**Fig. 2:**
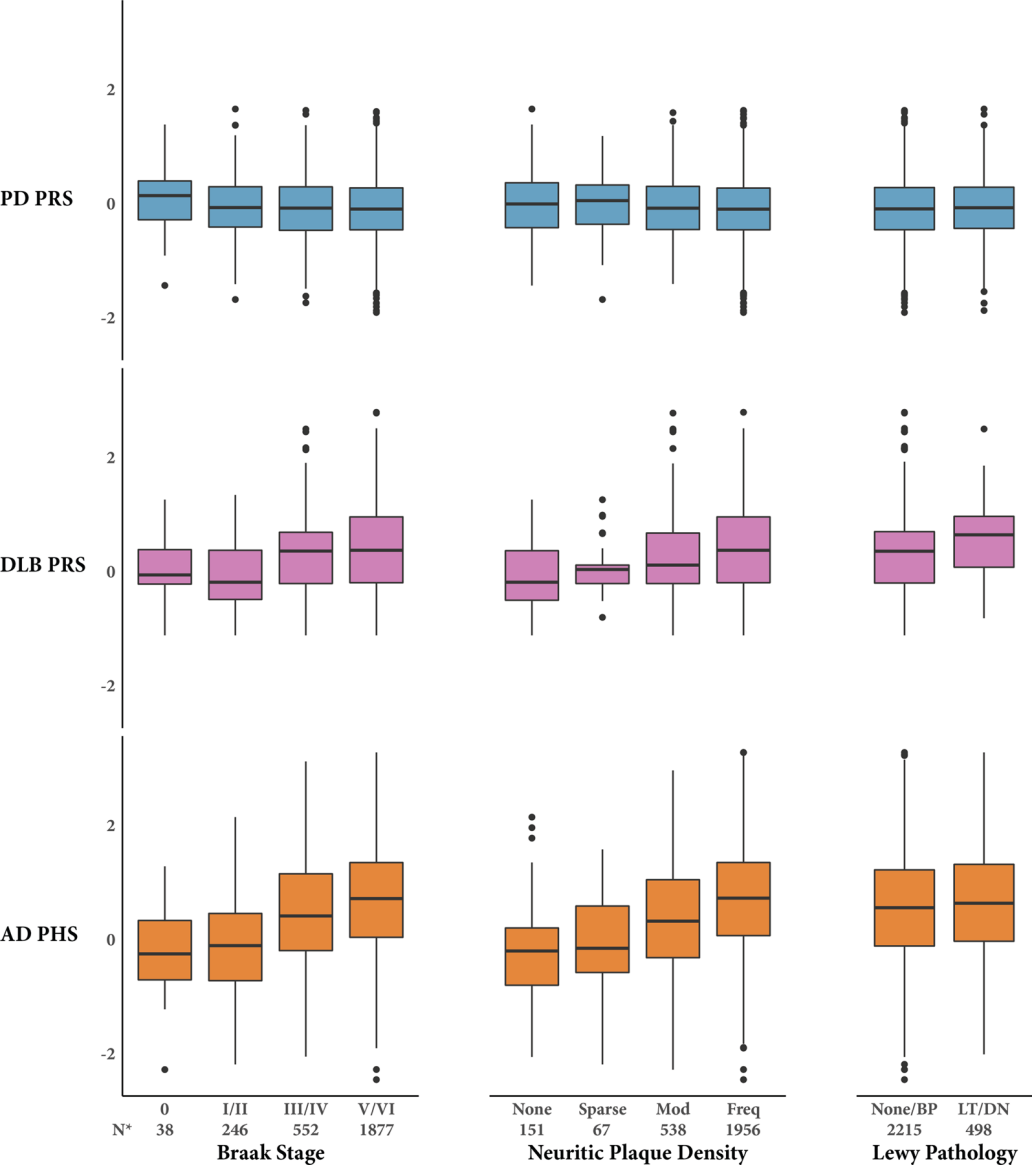
Relationship between polygenic scores and pathological variables. Graphical visualization of the relationship between the PD, DLB, and AD polygenic scores and measures of AD (Braak stage and neuritic plaque density) and Lewy pathology. Results of ordinal and binary logistic regression models for each polygenic score and pathological outcome variable are included in Table 3. *The number of participants is reported for the AD PHS and PD PRS analyses. The DLB PRS was analyzed in a subset of 2282 total participants. Abbreviations: PD, Parkinson’s disease, PRS, polygenic risk score; DLB, dementia with Lewy bodies; AD, Alzheimer’s disease; PHS, polygenic hazard score; BP, brainstem predominant; LT, limbic (transitional); DN, diffuse neocortical

The odds of having tau pathology at or above a given Braak stage increased with the AD PHS (*p* < .001, Table 3; Fig. 2). The PD PRS was not associated with Braak stage. Beginning with Braak stage II, the odds of having tau pathology at or above a given Braak stage increased with DLB PRS (*p* < .001). Without the *APOE* ε4 or ε2 dosage weights, we observed similar results for the association between Braak stage and the AD PHS (OR 1.20 95% CI [1.08–1.34]). Yet, without the *APOE* component weight, the DLB PRS was not associated with Braak stage (0.89 [0.73–1.08]).

The odds of having neuritic plaques at or above a given density increased with both the AD PHS and DLB PRS (*p* < .001, Table 3; Fig. 2). The PD PRS was not associated with neuritic plaque density. Without the *APOE* ε4 or ε2 dosage weights, we observed similar results for the association between the AD PHS and neuritic plaque density (OR 1.25 95% CI [1.10–1.43]). Conversely, without the *APOE* component weight, the odds of having neuritic plaques at or above a given density decreased with increasing DLB PRS (OR 0.76 [0.60–0.96]).

None of the polygenic scores were associated with the presence of at least limbic (transitional) LB pathology (Table 3; Fig. 2). Similarly, without the *APOE* ε4 or ε2 dosage weights, the AD PHS was not associated with the presence of at least limbic (transitional) LB pathology (OR 1.04 95% CI [0.89–1.22]). However, without the *APOE* component weight, the DLB PRS was associated with the odds of having at least limbic (transitional) LB pathology (3.41 [1.93–6.03]).

## Discussion

We tested whether genetic risk for AD, DLB, or PD may be useful to clinically distinguish between AD and DLB. The AD and DLB polygenic scores were associated with their respective pathological diagnostic categories, though not exclusively. We replicated the finding of an overrepresentation of the *APOE* ε4 allele in the pure DLB group compared to controls [24], but found that the ε4 allele frequency was higher in all AD groups (AD, AD + DLB, or AD + MTLS) than in the DLB group. Given this increased frequency and the strong, dose-dependent risk of the ε4 allele in AD, the inclusion of an *APOE* weight in the DLB PRS diminished its ability to specifically predict LB pathology. When the *APOE* weight was removed, the DLB PRS was associated with only DLB and AD + DLB pathological diagnosis groups, and was associated with increased LB but not AD pathology.

However, the dose-dependent weighting of the *APOE* ε4 allele is an important feature of the AD PHS, and enabled the distinction between AD groups and the non-AD groups. When removing the *APOE* ε2 and ε4 dosage weights, the AD PHS maintained its association with Braak stage and neuritic plaque density, but it also maintained associations with all pathological groups relative to the control group. The AD PHS includes genes associated with multiple biological processes implicated in AD, such as inflammation, synaptic function, and epigenetic regulation [10]. The associations suggest such processes may also be disrupted in non-AD dementias.

The PD PRS was associated with a clinical PD diagnosis, but not any pathological diagnosis or variable examined in this study. These results align with recent work finding genetic risk for PD explained only a small amount (.37%) of variance in DLB [7].

Despite striking group-level results, one limitation to the clinical utility of this work is the amount of individual variation in polygenic scores within pathological groups. It remains unclear whether assessing polygenic risk for AD and DLB in combination with clinical features and biomarkers improves the accuracy of clinical diagnoses at the individual level. Further, the polygenic scores used in these analyses were predominantly derived and tested on individuals of European ancestry. Known differences in genetic risk across racial and ethnic groups [17, 18, 23] suggest these findings may not generalize. Future work is required to develop polygenic scores in diverse populations.

## Conclusions

Despite few identified genome-wide significant variants, the DLB PRS without the *APOE* component weight was specifically associated with LB pathology and a pathological diagnosis of DLB, either alone or in combination with AD. The AD PHS was associated with ADNC but not LB pathology, and most strongly predicted a pathological diagnosis of AD, either alone or in combination with DLB or MTLS. Together, these results and the lack of associations with the PD PRS align with evidence that genetic risk for DLB is not simply situated in the middle of an AD-PD continuum, but has a distinct signature that can be exploited along with risk for AD to improve clinical diagnoses.

## Data Availability

The datasets supporting the conclusions of this article are available in the NACC (alz.washington.edu) or NIAGADS (niagads.org) repositories. A request for resources, materials, or participant referrals from the Shiley-Marcos ADRC can be made by emailing Christina Gigliotti, Ph.D at cgigliotti@ucsd.edu.

## Abbreviations

AD: Alzheimer’s disease
DLB: dementia with Lewy bodies
NC: neuropathologic changes
LB: Lewy body
PHS: polygenic hazard score
PD: Parkinson’s disease
*APOE*: apolipoprotein E
PRS: polygenic risk score
ADRC: Alzheimer’s Disease Research Center
UCSD: University of California, San Diego
NACC: National Alzheimer’s Coordinating Center
FTD: frontotemporal lobar degeneration
MTLS: medial temporal lobe sclerosis
CERAD: Consortium to Establish a Registry for Alzheimer’s Disease
ADGC: Alzheimer’s disease Genetics Consortium
NIAGADS: National Institute on Aging Genetics of Alzheimer’s disease Data Storage Site
SNPs: single-nucleotide polymorphisms
IGAP: International Genomics of Alzheimer’s Project
GWAS: genome-wide association study
ADNC: Alzheimer’s disease neuropathologic changes
